# In Systemic Sclerosis, a Unique Long Non Coding RNA Regulates Genes and Pathways Involved in the Three Main Features of the Disease (Vasculopathy, Fibrosis and Autoimmunity) and in Carcinogenesis

**DOI:** 10.3390/jcm8030320

**Published:** 2019-03-07

**Authors:** Marzia Dolcino, Elisa Tinazzi, Antonio Puccetti, Claudio Lunardi

**Affiliations:** 1 Department of Medicine, University of Verona, 37134 Verona, Italy; marziadolcino@gmail.com (M.D.); elisa.tinazzi@univr.it (E.T.); 2 Department of Experimental Medicine, Section of Histology, University of Genova, 16132 Genova, Italy; apuccetti@gmail.com

**Keywords:** systemic sclerosis, long non-coding RNA, signaling pathway, protein–protein (PPI) network, gene module

## Abstract

Systemic sclerosis (SSc) is an autoimmune disease characterized by three main features: vasculopathy, immune system dysregulation and fibrosis. Long non-coding RNAs (lncRNAs) may play a role in the pathogenesis of autoimmune diseases and a comprehensive analysis of lncRNAs expression in SSc is still lacking. We profiled 542,500 transcripts in peripheral blood mononuclear cells (PBMCs) from 20 SSc patients and 20 healthy donors using Clariom D arrays, confirming the results by Reverse Transcription Polymerase-chain reaction (RT-PCR). A total of 837 coding-genes were modulated in SSc patients, whereas only one lncRNA, heterogeneous nuclear ribonucleoprotein U processed transcript (ncRNA00201), was significantly downregulated. This transcript regulates tumor proliferation and its gene target hnRNPC (Heterogeneous nuclear ribonucleoproteins C) encodes for a SSc-associated auto-antigen. NcRNA00201 targeted micro RNAs (miRNAs) regulating the most highly connected genes in the Protein-Protein interaction (PPI) network of the SSc transcriptome. A total of 26 of these miRNAs targeted genes involved in pathways connected to the three main features of SSc and to cancer development including Epidermal growth factor (EGF) receptor, ErbB1 downstream, Sphingosine 1 phosphate receptor 1 (S1P1), Activin receptor-like kinase 1 (ALK1), Endothelins, Ras homolog family member A (RhoA), Class I Phosphoinositide 3-kinase (PI3K), mammalian target of rapamycin (mTOR), p38 mitogen-activated protein kinase (MAPK), Ras-related C3 botulinum toxin substrate 1 (RAC1), Transforming growth factor (TGF)-beta receptor, Myeloid differentiation primary response 88 (MyD88) and Toll-like receptors (TLRs) pathways. In SSc, the identification of a unique deregulated lncRNA that regulates genes involved in the three main features of the disease and in tumor-associated pathways, provides insight in disease pathogenesis and opens avenues for the design of novel therapeutic strategies.

## 1. Introduction

Systemic Sclerosis (SSc) is a rare autoimmune disease characterized by endothelial disfunction, dysregulation of the immune system and increased extracellular matrix deposition leading to extended involvement and fibrosis of skin and internal organs and resulting in a remarkable heterogeneity in clinical features and in disease course [1].

Several factors have been shown to contribute to the onset of the disease, such as genetic susceptibility, environmental factors including viral infections [2,3] and epigenetic mechanisms, such as microRNAs (miRNAs) [4,5].

We have already reported the gene expression profiling of peripheral blood mononuclear cells (PBMCs) obtained from patients affected by SSc [6] showing the presence of cancer-related gene signatures.

Indeed, an increased frequency of different types of cancer, including breast, lung, and hematologic malignancies in SSc has been already described [7], and has been associated with the presence of particular autoantibodies [8]. In addition, SSc may also be a paraneoplastic disease [9,10] suggesting a strong link with cancer.

In this study we focused our attention on the epigenetic mechanisms that may be involved in SSc pathogenesis by analyzing the expression profiles of long non-coding RNAs (lncRNAs) in SSc patients.

For the first time, we propose an integrated analysis of lncRNAs, miRNAs and gene expression profiles in SSc patients leading to the identification of lncRNAs modulated in the disease. We identified a lncRNA involved both in pathogenetically relevant molecular pathways of the disease and in tumor-associated pathways, strongly associated to the disease.

## 2. Materials and Methods

### 2.1. Patients

Twenty patients affected by SSc, attending the Unit of Autoimmune Diseases at the University Hospital of Verona, Northern Italy, and 20 sex and age matched healthy controls were enrolled. Both patients and controls were subjects of Caucasian origin from Northern Italy. All patients were diagnosed accordingly to the American College of Rheumatology (ACR)/European League Against Rheumatism (Eular) classification criteria for SSc [11]. The distinction between limited (lSSc) and diffuse cutaneous SSc (dSSc) was performed following the criteria proposed by Le Roy et al. [12]. Ten patients were affected by lSSc and 10 by dSSc. The clinical and demographic features of the patients enrolled in the study are reported in Table 1.

Samples obtained from 10 patients with lSSc, 10 patients with dSSc and 20 healthy controls were used for the gene and lncRNAs expression analysis. Blood samples were collected from patients with active disease (European Scleroderma Study Group activity index > 3) [13] within 24 months after diagnosis and in the absence of immunosuppressive therapies. Samples were processed immediately after the blood draw.

Twenty more patients and 20 healthy controls have been analyzed as validation groups (Supplementary Table S1).

A written informed consent was obtained by all the participants to the study and the study protocol was approved by the Ethical Committee of the Azienda Ospedaliera Universitaria Integrata di Verona. All the investigations have been performed according to the principles contained in the Helsinki declaration.

### 2.2. Microarray Analysis

Blood samples collection was performed using BD (Becton Dickinson)Vacutainer K2EDTA tubes and 21-gauge needles.

PBMCs were isolated by Ficoll–HyPaque (Pharmacia Biotech, Quebec, Canada) gradient centrifugation. The PBMCs distribution was similar between patients and controls. Total RNA extraction from PBMCs (10^7^ cells) was achieved with miRNeasy mini kit (Qiagen GmbH, Hilden, Germany). cRNA preparation, samples hybridization and scanning were carried out following the Affymetrix (Affymetrix, Santa Clara, CA, USA) provided protocols, by Cogentech Affymetrix microarray unit (Campus IFOM IEO, Milan, Italy). All samples were hybridized on Human Clariom D (Thermo Fisher Scientific, Waltham, MA, USA) gene chip. Signal intensities were analyzed with the Transcriptome Analysis Console (TAC) 4.0 software (Applied Biosystem, Foster City, CA USA by Thermo Fisher Scientific).

The Human Clariom D arrays interrogates more than 540,000 human transcripts starting from as little as 100 pg of total RNA. Signals intensity was background-adjusted, normalized, and log-transformed using the Signal Space Transformation (SST)-Robust Multi-Array Average algorithm (RMA).

Differentially expressed genes that showed an expression level at least 1.5 fold different in the test sample versus control sample at a significant level (*p* ≤ 0.01) were chosen for final consideration. *p*-values were calculated applying the unpaired *t*-test and Bonferroni multiple testing correction.

Targets annotations of ncRNA00201 were retrieved using starBase v2.0 (http://starbase.sysu.edu.cn/) where lncRNAs interactions, experimentally validated by high-throughput experimental technologies, are registered [14].

The list of Gene targets of microRNAs (miRNAs) that are targeted by ncRNA00201 were obtained from the FunRich database (www.funrich.org/) [15].

### 2.3. Protein–Protein Interaction (PPI) Network Construction and Network Clustering

The PPI network was constructed upon the experimentally validated protein–protein interactions using STRING (Search Tool for the Retrieval of Interacting Genes) version 10.5 (http://string-db.org/) [16].

Network topological analysis was performed using the Cytoscape software (version 3.4.0) (www.cytoscape.org) [17].

High-flow areas (highly connected regions) of the network (modules) were detected using the MCODE plugin of Cytoscape (k-core = 4 and node score cutoff = 0.2).

### 2.4. Gene Functional Classification and Enrichment Analysis

Functional classification of genes into Biological Processes (BPs) were performed according to the Gene Ontology (GO) annotations (www.geneontology.org) using Panther expression analysis tools (version 14.0) (http://pantherdb.org/) [18].

Pathways classification and enrichment (hypergeometric *p*-value ≤0.05) analysis were performed with FunRich (version).

### 2.5. Real Time PCR of ncRNA00201

A total of 500 ng of total RNA was treated with 1 unit of DNase I Amplification Grade (Invitrogen; Carlsbad, CA, USA.). First-strand cDNA was generated using the SuperScript IV First-Strand Synthesis System (Invitrogen; Carlsbad, CA, USA.) with random hexamers, according to the manufacturer’s protocol. Real time PCR was performed in triplicate with PowerUp™ Sybr^®^ Green reagent (Applied Biosystems; Foster City, CA, USA) in a QuantStudio 6 Flex system (Applied Biosystems; Foster City, CA, USA). Relative expression levels were calculated for each sample after normalization against the geometric mean of the housekeeping genes GAPDH and beta-actin (ACTB) expression. The ΔΔCt method was used for comparing relative fold expression differences. The data are expressed as fold changes with respect to healthy. The primers used for the detection were: GCA GGA GAA TCG CTT GAA C (forward) and ACC CTA CCA TCC AAC TTC AC (reverse).

### 2.6. Real Time PCR of Genes Modulated in SSc Patients

First-strand cDNA was generated using the SuperScript III First-Strand Synthesis System for Reverse transcription Polymerase-chain Reaction (RT-PCR) Kit (Invitrogen), with random hexamers, according to the manufacturer’s protocol. PCR was performed in a total volume of 25 μL containing 1× Taqman Universal PCR Master Mix, no AmpErase UNG and 2.5 μL of cDNA; pre-designed, Gene-specific primers and probe sets for each gene were obtained from Assay-on-Demande Gene Expression Products (Applied Biosystems).

Real Time PCR reactions were carried out in a two-tube system and in singleplex. The Real Time amplifications included 10 min at 95 °C (AmpliTaq Gold activation), followed by 40 cycles at 95 °C for 15 s and at 60 °C for one minute. Thermocycling and signal detection were performed with 7500 Sequence Detector (Applied Biosystems). Signals were detected according to the manufacturer’s instructions. This technique allows the identification of the cycling point where PCR product is detectable by means of fluorescence emission (Threshold cycle or Ct value). The Ct value correlates to the quantity of target mRNA. Relative expression levels were calculated for each sample after normalization against the housekeeping genes GAPDH, beta-actin and 18s ribosomal RNA (rRNA), using the ΔΔCt method for comparing relative fold expression differences. Ct values for each reaction were determined using TaqMan Software and Digital Store (SDS) analysis software (version 2.4.0). For each amount of RNA tested triplicate Ct values were averaged. Because Ct values vary linearly with the logarithm of the amount of RNA, this average represents a geometric mean.

### 2.7. Real Time PCR of microRNAs Targeted by ncRNA00201

miRNAs expression was assayed by TaqMan^®^ Advanced miRNA assays chemistry (Applied Biosystems, Foster City, CA, USA). Briefly, 10 ng of total RNA was reverse transcribed and pre-amplified with TaqMan^®^ Advanced miRNA cDNA synthesis kit following manufacturer’s instructions (Applied Biosystems, Foster City, CA, USA). Pre-amplified cDNA was diluted 1/10 in nuclease-free water and 5 µL of diluted cDNA for each replicate were loaded in PCR. 20 µL PCR reactions were composed by 2X Fast Advanced Master Mix and TaqMan^®^ Advanced miRNA assays for miR-30b-5p, miR-30e-5p, miR-302a-3p, miR-520d-3p, miR-613 and miR-9-5p. The mean of Ct for hsa-miR-16-5p and hsa-miR-26a-5p expression was used to normalize miRNA expression. Real time PCR were carried out in triplicate on a QuantStudio 6 Flex instrument (Applied Biosystems, Foster City, CA, USA). Expression values were reported as fold change with respect to healthy controls by ΔΔCt method using QuantStudio Real-Time PCR system software v. 1.3.

### 2.8. Detection of Soluble Mediators in Sera of SSc Patients

Serum levels of interleukin-6 (IL-6), macrophage migration inhibitory factor (MIF) selectin P (SELP) chemokine C-X-C motif ligand 10, (CXCL10) and basigin (BSG) were detected using commercially available Enzyme-Linked Immunosorbent Assay (ELISA) kits that were supplied by Aviva Systems Biology (IL-6; cat. n. OKDB00046), R&D (MIF; cat n. DMF00B and SELP; cat. n. DPSE00), Life Span Biosciences (LSBio) (CXCL10; cat. n. LS-F586-1) and Creative Diagnostics (BSG; cat n. DEIA2873).

## 3. Results

With the aim of identifying lncRNAs potentially involved in SSc pathogenesis, the expression of more than 540,000 human transcripts, including those ascribed to more than 50,000 lncRNAs was profiled at the same time, in a cohort of 40 PBMCs samples (20 SSc and 20 healthy subjects). Transcriptional profiles of SSc patients and healthy subjects were compared and, after a robust filtering procedure (Bonferroni-corrected *p*-value ≤ 0.01 and fold change ≥ |1.5|), 837 coding-genes were significantly modulated (Supplementary Table S2). More than one hundred lncRNAs were detectable, but only one, long non-coding heterogeneous nuclear ribonucleoprotein U antisense RNA 1 (HNRNPU-AS1, also known as ncRNA00201) satisfied the above mentioned criteria (statistical and fold change) showing a significant modulation (Table 2).

ncRNA00201 has been showed to be overexpressed in pancreatic ductal adenocarcinoma (PDAC) tissues and cell lines, and plays a role in the regulation of cell proliferation, invasion, and migration. Indeed, the knockdown of this lncRNA reduces proliferation and in vitro migration and invasion of PDAC cell lines [19].

The functional classification by Gene Ontology (http://www.geneontology.org/) of the 837 differentially expressed genes (DEGs) revealed the presence of a large number of differentially expressed coding genes involved in biological processes (BPs) and signaling pathways that are strictly connected to SSc pathogenesis, including, immune response BP, interferon alpha/beta signaling, IL-6 mediated signaling events BP, inflammatory response BP, apoptosis BP, angiogenesis BP, Vascular Endothelial Growth Factor (VEGF) and VEGFR signaling, cell adhesion BP, blood coagulation BP, endothelin signaling, PDGFR-beta signaling, EGF receptor (ErbB1) signaling, TGF-beta receptor signaling, extracellular matrix (ECM) component and organization, proteoglycan syndecan mediated signaling events, and positive regulation of fibroblast proliferation BP.

Table 2 shows a selection of DEGs involved in these meaningful biological processes and pathways.

The modulation of genes involved in immune response highlighted the immune system alterations that characterize SSc and other autoimmune diseases. Not surprising, in SSc samples we found upregulation of genes belonging to the type alpha and beta interferon signaling pathways, that is a common trait of autoimmune diseases and, this finding has also been previously overserved in SSc PBMCs [20]. In addition, we found a strong upregulation of interleukin-6 (IL-6) (F.C. 12.69) a cytokine involved in inflammatory and autoimmune diseases including SSc and, besides that, we found overexpression of several members of its signaling pathway (see Table 2).

The presence of modulated genes involved in the inflammatory process reflected the chronic inflammation that accompanies SSc. Moreover the upregulation of genes involved in apoptosis is not surprising, since apoptosis is the first event of vascular damage in early SSc [2]. The modulation of genes involved in angiogenesis gives evidence of the angiogenic response to tissue ischemia and vascular damage that occurs in the initial stages of SSc. We also have to mention that, members of the proangiogenetic VEGF and VEGFR signaling pathway were modulated in SSc (see Table 2).

The vascular response to endothelial damage consists of endothelial activation with increased expression of adhesion molecules and, indeed, several adhesion molecules were overexpressed in SSc samples.

As it is well known, the spectrum of vascular abnormalities that characterizes SSc also involves disorders of the blood coagulation-fibrinolysis system [21] and, noteworthy, we found upregulation of several genes involved in blood coagulation.

The earliest vascular changes in SSc also include a dysregulated control of vascular tone and indeed we have to mention that endothelins are among the strongest vasoconstrictors known, and are involved in vascular diseases of several organs. Interestingly in SSc samples several components of the endothelins pathway were overexpressed.

Moreover, we found modulation of members of the platelet derived growth factor receptor-beta (PDGFR-B). PDGFR-B has been implicated in SSc [22] and it plays a role in activation of the epidermal growth factor receptor (EGFR) that is also involved in the disease. In particular, in scleroderma fibroblasts, aberrant activation of EGF-mediated signaling pathways, can upregulate the receptor II of transforming growth factor beta (TGFBRII) [23], the growth factor primary playing a role in the development of connective tissue fibrosis that is typically involved in SSc pathogenesis and, noteworthy, members of the EGFR pathway were modulated in SSc. Moreover the deregulation of both PDGF and EGF pathways has been already described in a previous study on gene expression profiles in SSc peripheral blood [24].

Consistently with these findings, other modulated genes were involved in the signaling of the profibrotic cytokine TGF-beta.

Fibrosis is supported by extracellular matrix (ECM) remodeling and, consistently, we found overexpression of ECM components and of molecules involved in ECM organization. Moreover, we found an increased expression of molecules involved in proteoglycan syndecan-mediated signaling events (see Table 2) and of genes involved in the positive regulation of fibroblasts proliferation.

The expression levels of ncRNA00201, selected miRNAs and coding genes were validated by RT-PCR in the entire series of the analyzed patients and in an expanded panel of SSc patients (20) and healthy controls (20 subjects) (Supplementary Table S1). Significantly different expression levels were found for all tested transcripts as compared to healthy controls (Supplementary Figures S1 and S2).

Gene expression results were also confirmed by the detection of several soluble mediators (including interleukin-6, IL-6; macrophage migration inhibitory factor, MIF; selectin P, SELP; chemokine C-X-C motif ligand 10, CXCL10 and basigin, BSG) in the sera of SSc patients. As shown in Supplementary Figure S3 the serum levels of all the tested molecules were significantly different in SSc patients when compared to healthy donors.

Noteworthy, the pathways enrichment analysis confirmed that the important signaling networks described in Table 2 were all included in the list of pathways significantly (*p* < 0.05) enriched in the 837 genes, along with others that were likewise connected to autoimmune and inflammatory response, vascular damage (i.e., apoptosis, platelet activation and aggregation, Urokinase-type plasminogen activator (uPA) and uPAR-mediated, Thrombin/protease-activated receptor (PAR) signaling etc.) and fibrosis (i.e., glypican, syndecan-3 mediated, regulation of nuclear Small Mother Against Decapentaplegic (SMAD) 2/3 signaling etc.) (Supplementary Table S3).

Since modulated genes were well representative of the main features of the disease, we decided to verify if the only modulated lncRNA could be functionally connected to SSc transcriptome, thus playing a role in SSc pathogenesis. To this purpose we extracted the complete list of experimentally validated genes and microRNAs (miRNAs) targets of ncRNA00201, and we found that 56 miRNAs and 31 genes were annotated as target of ncRNA00201 (Supplementary Table S4). One of the gene targets, named hnRNPC, belonged to a subfamily of ubiquitously expressed heterogeneous nuclear ribonucleoproteins (hnRNPs) and interestingly encoded for a known antigen identified in SSc [25].

To dissect all the possible connections between ncRNA00201 and the SSc transcriptome we analyzed the lists of genes targeted by each of the 56 miRNAs (3759 genes) selecting only transcripts that also resulted significantly modulated on the array. Among the 3759 gene targets, 138 were modulated in SSc patients and were further analyzed along with their targeting miRNAs (47/56) (Supplementary Table S5). We therefore selected only those miRNAs that targeted genes with evidence of modulation in SSc patients to bona fide outline authentic interactions that are most probably established in SSc.

The analysis of signaling pathways, in which the 138 DEGs may be involved, showed that these genes were present in the 83% (147/176) of pathways significantly enriched in the SSc transcriptome (Supplementary Table S6).

A protein–protein interaction (PPI) network including all the protein products of the 837 modulated genes that showed experimentally validated interactions was constructed and, the obtained network included 693 nodes (interacting partners) and 2226 edges (interactions), showing a good PPI enrichment *p*-value (<10^−16^) indicating that the network contained significantly more interactions than expected. In other words, the connected proteins had more interactions among themselves than what would be expected for a random set of proteins of similar size, drawn from the genome. Such an enrichment indicates that these proteins are biologically connected, as a group.

**Figure 1:**
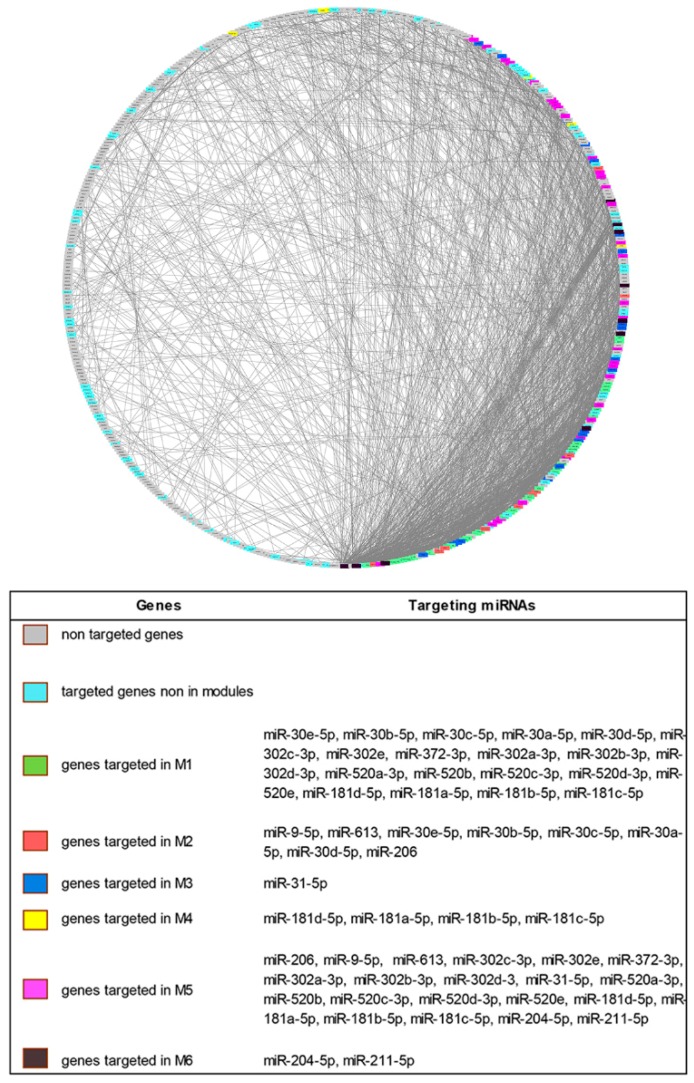
Degree Sorted Circle Layout of the Protein–Protein Interaction (PPI) network of differentially expressed genes in Systemic Sclerosis patients. Nodes are ordered around a circle based on their degree of connectivity (number of edges). Genes not targeted, genes targeted but not included in modules and genes targeted and included in the modules are differently colored.

The topological analysis of the PPI network showed the presence of a large number of targeted genes in areas with high density of connections (Figure 1).

When a modular analysis was performed, we could identify six areas of the PPI network in which the most highly connected genes were present (modules).

Since the modulation of highly connected genes is expected to have a wider impact on the transcriptome than the targeting of several isolated genes, we checked if ncRNA00201 could target genes included in the modules and, noteworthy, we observed that genes modulated by miRNA targets of ncRNA00201 were present in each of the 6 modules (Supplementary Table S7).

To dissect the most relevant interactions through which ncRNA00201 could influence the pathogenesis of the disease, the miRNAs targeted by ncRNA00201 were filtered, prioritizing those that targeted at least 7 genes in the whole SSc transcriptome (i.e., the 837 modulated genes) and at least two modules. By these criteria, 26 miRNAs target of ncRNA00201 were selected. Table 3 shows the 26 selected miRNAs and summarizes their targeted genes in the SSc transcriptome and in the modules.

Interestingly, among the selected miRNAs, miR-30b-5p and miR-31-5p were previously described as deregulated in SSc whereas, all the 26 miRNAs are modulated in several types of human cancer (Table 3).

The selected miRNAs targeted module-associated-genes with important biological significance, indeed these genes were involved in inflammation, in the modulation of immune response regulating signaling, in maintaining vascular homeostasis, in tissue remodeling, and also in metastasis development in many cancers.

Indeed, Module 1 (M1) was targeted by 20 miRNAs (see Table 3) that globally modulated SOCS3, UBE2F, UBE2J1, IRF9, and CBLB. SOCS3, a major regulator of inflammation, is rapidly induced by JAK-STAT (Janus kinase-signal transducer and activator of transcription) signaling during the inflammatory response. UBE2F and UBE2J1 are ubiquitin conjugating enzymes that are involved in inflammation and in metastasis development in many cancers [54]. IRF9 is strongly induced by type I interferons and CBLB is involved in the regulation of immune response modulating signaling by both T-cell and B-cell receptors [55].

M2 was targeted by eight miRNAs including the members of the miRNA 30s family (miR-30a, 30b, 30c, 30d, and 30d-5p miRNAs) that targeted GNAI2 and miR-206, miR-9-5p and miR-613 that modulated CXCL11. GNAI2 plays a role in chemokine receptor signaling in B-cells [56] and CXCL11 is a proinflammatory molecule whose serum levels are increased in SSc [57].

M3 was targeted by miR-31-5p that modulated KDELR2. This molecule is involved in extracellular matrix degradation and in the signaling of the Src family proteins that is activated during cell invasion leading to metastasis development [58].

In M4 the gene APIS3 was targeted by members of the miR-181s family (including miR-181-a/b/c/d-5p). Lacking of AP1S3 gene has been associated to the development of skin inflammation [59] and interestingly, AP1S3 was down-modulated in SSc samples (see Supplementary Table S1).

M5 was the most targeted module since it was targeted by 21 out of the 26 selected miRNAs (see Table 3). In this module 5 genes were targeted including GCH1, GUCY1A3, PAX5, SPARC and BCL2. GCH1, also named GTPCH1 is implicated in the synthesis of the three nitric oxide synthases (NOS1–3) [60] and endothelial cell GCH1-dependent endothelial nitric oxide (eNOS) regulation plays an important role in maintaining vascular homeostasis [61]. GUCY1A3 encodes for the α1 subunit of nitric oxide (NO) receptor and is involved in the control of smooth muscle cells relaxation [62].

PAX5 is indispensable for the B-cell lineage specification and maintenance [63] and has been implicated in human B cell malignancies, including acute lymphoblastic leukemias and non-Hodgkin lymphomas [64].

SPARC (also known as osteonectin) is a glycoprotein of the extracellular matrix expressed in concomitance with tissue remodeling and wound repair. Interestingly SPARC upregulation has been described in cultured dermal fibroblasts from patients with SSc [65] and it has been observed that SPARC silencing can reduce collagens overproduction in SSc skin fibroblasts [66].

The BCL2 gene is involved in apoptosis and its co-expression with the oncogene MYC is associated with a poor prognosis in B-cell lymphoma patients [67]. Interestingly both BCL2 and MYC were upregulated in SSc patients (see Supplementary Table S1).

In module M6, PSME1 was targeted by miR-204-5p and miR-211-5p. PSME1, also named PA28, is a proteasome component that influences antigen processing. Proteasomal system plays crucial roles in important biological processes including cell differentiation, proliferation, apoptosis, transcriptional activation and angiogenesis, and is a pivotal target for treatment of several diseases including autoimmune diseases and cancer [68].

**Figure 2:**
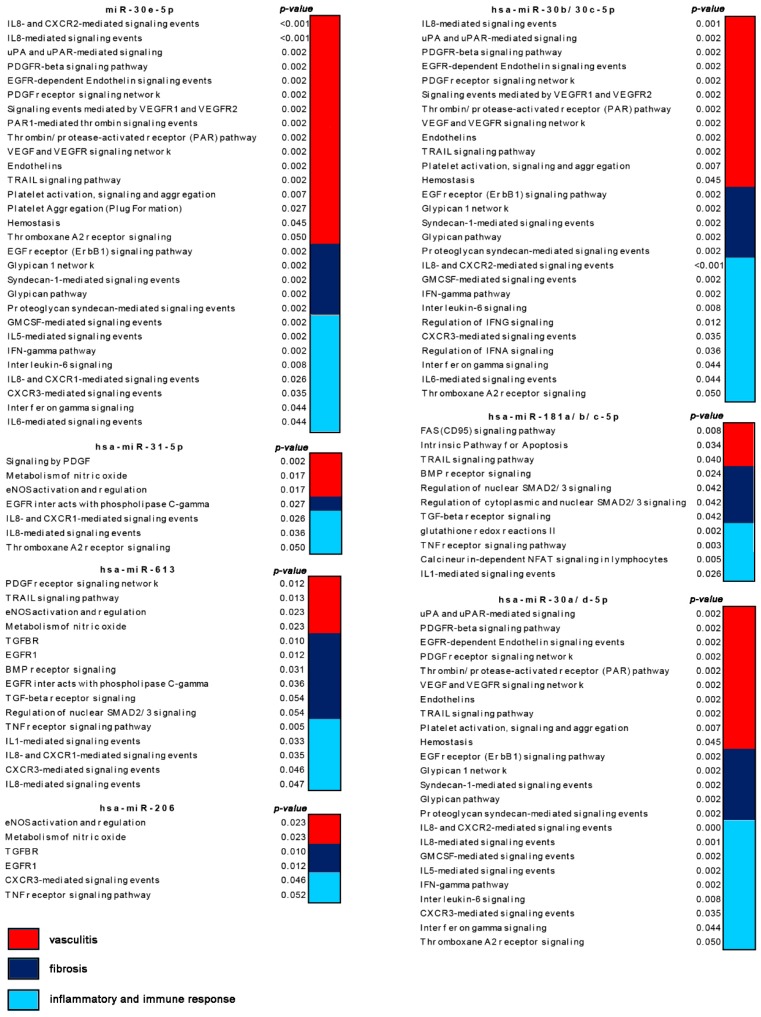
Schematic representation of enriched (*p* < 0.05) signaling pathways modulated by selected micro RNAs (miRNAs) that are targeted by non-coding RNA 201 (ncRNA00201). Pathways are grouped by their relevance to the disease features including immune and inflammatory response, vasculitis and fibrosis.

We also observed that a large proportion of the pathways enriched (*p* < 0.05) in modulated genes targeted by the 26 selected miRNAs were linked to the three main features of the disease (i.e., immune and inflammatory response, vasculitis and fibrosis) thus confirming their possible involvement in SSc pathogenesis. Figure 2 shows a selection of the above mentioned enriched signaling pathways in which are involved modulated genes targeted by 11 of the 26 selected miRNAs.

**Figure 3:**
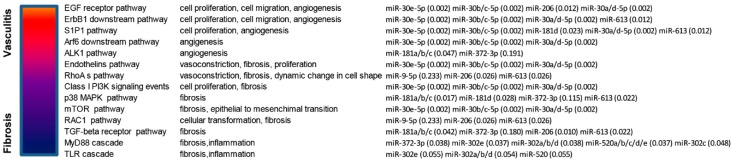
Schematic representation of enriched (*p* < 0.05) signaling pathways modulated by selected miRNAs that are targeted by ncRNA00201. Selection of enriched pathways that are both involved in Systemic Sclerosis (SSc) and in cancer development. Enrichment *p*-values are showed in brackets.

Interestingly, several of the aforementioned pathways are also deregulated in many forms of cancer. These pathways included for example EGF receptor, ErbB1 downstream, Sphingosine-1-phosphate receptor 1 (S1P1), Arf6 downstream, ALK1, Endothelins, RhoA, Class I PI3K (Phosphatidylinositol 3-kinase), mTOR, p38 MAPK, RAC1, TGF-beta receptor, MyD88 and Toll-like receptors signaling pathways (Figure 3).

We thereafter aimed at identifying modulated genes targeted by the selected miRNAs whose deregulation could have the widest impact on the global disease-associated gene modulation. We therefore narrowed our analysis to modulated genes targeted by the 26 miRNAs that were included in the six modules and we identified five transcripts, namely IRF9, GUCY1A3, SOCS3, BCL2 and GNAI2 that were targeted by the highest number of selected miRNAs (see Table 2).

Interestingly, we observed that they were associated to a large number of pathways (136) and that 82 (60%) of such pathways are significantly enriched (*p* < 0.05) in the whole SSc transcriptome such as for example interferon alpha/beta, apoptosis, endothelins, PDGF receptor beta, TGF-beta receptor and regulation of SMAD (Supplementary Table S8).

## 4. Discussion

Though it has been widely recognized that lncRNAs play a pivotal role in the regulation of autoimmune diseases [69], an extensive analysis of lncRNAs in SSc is still lacking. We therefore performed a comprehensive analysis assessing the expression profiles of a very large number of lncRNAs in SSc patients, and we could find that, differently from what we had already observed in other autoimmune diseases [70], a single lncRNA, namely ncRNA00201, appeared to be significantly modulated in SSc.

Among the gene targets of ncRNA00201, there is the hnRNPC, which encodes for a known autoantigen in SSc [25].

Interestingly, ncRNA00201 has been showed to be involved in cancer proliferation [19] and this finding may reinforce the hypothesis of a link between SSc and tumor development, as we have recently suggested [6].

By a combined analysis of the expression profiles of a vast number of coding and non-coding transcripts, we have been able to observe that ncRNA00201 alone controls biological processes and pathways closely related to the three main features of SSc, including immune and inflammatory response, vasculitis and fibrosis.

We moreover identified 26 miRNAs by which ncRNA00201 could modulate the expression of several genes crucial to the disease, thus highlighting the most effective lncRNA-miRNA-gene interactions in SSc. Interestingly, all the 26 miRNAs are modulated in several types of human cancer.

We also showed that ncRNA00201 is able to modulate pathways that are associated to both SSc and tumor development such as EGF receptor, ErbB1 downstream, S1P1, Arf6 downstream, ALK1, Endothelins, RhoA, Class I PI3K, mTOR, p38 MAPK, RAC1, TGF-beta receptor, MyD88 and Toll-like receptors signaling pathways, confirming our previous observations [6].

Indeed, changes in EGFR expression and autoantibodies to this receptor have been observed in patients with SSc [71], nevertheless there is evidence for the involvement of EGFR signaling in the development of different carcinoma types [72].

The activation of S1P signaling pathway can induce many of the alterations observed in SSc patients [73], moreover, S1P receptors regulate cell proliferation in various tumors [74].

Arf6 is involved in angiogenesis [75] and is implicated in tumor metastasis [76]. ALK1 inhibition reduces pro-fibrotic genes expression in SSc fibroblasts [77] moreover, this gene is an emerging target for antiangiogenic therapy in cancer [78]. Endothelins (ET) play important roles in the in the fibrotic process associated to SSc and have a documented role in the development of cancer [79]. RhoA signaling, is frequently altered in many types of human tumors [80] and the expression of RhoA is elevated in SSc endothelial cells [81].

PI3K signaling is involved in collagen gene expression and in the antiapoptotic phenotype that characterizes SSc fibroblasts [82,83]. This pathway acts in synergy with the mTOR signaling in the development of many cancers [84]. Interestingly, mTOR blockade can reduce the pro-fibrotic action of TGF-beta and its therapeutic implications for SSc have been suggested [85]. 

The p38 MAPK and the Rac1 signaling were also associated to SSc fibrosis [86,87] and to cancer progression [88,89].

Finally, other signaling typically involved in both SSc and cancer development are TGF-beta receptor and MyD88/Toll-like receptors pathways [90,91,92,93].

In conclusion, this study is the first report of a unique lncRNA that controls gene networks that may contribute to SSc pathogenesis and that may play a predisposing role to the well documented development of malignancies in SSc.

Finally, we may propose that ncRNA00201 may be a potential target in the development of novel therapeutic strategies.

## Figures and Tables

**Table 1:** Demographic and clinical and features of patients and healthy subjects enrolled in the study.

Demographic and Clinical Features of Systemic Sclerosis Patients and Healthy Controls			
Healthy controls		20	
	Male/Female	3/17	
	Mean age (years)	55 ± 11	
Patients		lSSc	dSSc
		10	10
	Male/Female	2/8	1/9
	Mean age (years)	57 ± 13	55 ± 10
Laboratory findings	ANA	9 (90%)	10 (100%)
	Anti-centromere	6 (60%)	2 (20%)
	Scl-70	0	8 (80%)
Lung involvement	Interstitial disease	3 (30%)	6 (60%)
	Pulmonary arterial hypertension	2 (20%)	1 (10%)
Skin involvement	mRSS	8 ± 3	14 ± 8
	Digital ulcers	3 (30%)	5 (50%)
Video Capillaroscopy	Early	2 (20%)	3 (30%)
	Active	5 (50%)	4 (40%)
	Late	3 (30%)	3 (30%)
Kidney involvement		0 (0%)	1 (10%)
Gastro-intestinal involvement		7 (70%)	9 (90%)

lSSc = limited Systemic Sclerosis; dSSc = diffuse Systemic Sclerosis; ANA = anti-nuclear antibody; Scl-70 = Sclero-70; mRSS = modified Rodnam Skin Score.

**Table 2:** Selection of significantly differentially expressed genes and lncRNA in Systemic Sclerosis patients versus healthy subjects.

ID	Fold Change	*p*-Value	Gene Symbol	Description	Accession Number
TC0100018570.hg.1	−1.8	0.0003	ncRNA00201	heterogeneous nuclear ribonucleoprotein U antisense RNA 1	ENST00000366527.3
**Apoptosis**					
TC0100013000.hg.1	1.57	0.0012	CASP9	caspase 9	AB020979.1
TC1000007990.hg.1	2.32	0.0017	DDIT4	DNA damage inducible transcript 4	NM_019058.3
TC1900011729.hg.1	2.1	<0.0001	TIMM50	translocase of inner mitochondrial membrane 50 homolog	NM_001001563.3
TC0600014045.hg.1	1.65	0.0064	PDCD2	programmed cell death 2	NM_002598.3
TC1800008891.hg.1	2.1	0.0013	BCL2	B-cell CLL/lymphoma 2	NM_000633.2
TC0200010972.hg.1	1.68	0.0003	MFF	mitochondrial fission factor	NM_001277061.1
TC1500008231.hg.1	1.81	0.0002	AEN	apoptosis enhancing nuclease	NM_022767.3
TC0500010104.hg.1	1.63	0.0006	DAP	death-associated protein	NM_001291963.1
**Immune Response**					
TC0300013859.hg.1	2.95	0.0015	CD200	CD200 molecule	NM_005944.6
TC2000007519.hg.1	1.59	0.009	CD40	CD40 molecule, TNF receptor superfamily member 5	NM_001250.5
TC1900008166.hg.1	3.55	0.0029	CD79A	CD79a molecule, immunoglobulin-associated alpha	L32754.1
TC1700011430.hg.1	2.61	0.0011	CD79B	CD79b molecule, immunoglobulin-associated beta	KM057839
TC0700009461.hg.1	2.77	0.0008	TRBV24-1	T cell receptor beta variable 24-1	AY373826
TC0600007597.hg.1	1.59	0.0039	LST1	leukocyte specific transcript 1	NM_007161
TC1600011368.hg.1	2	0.0002	LAT	linker for activation of T-cells	AF036905
TC1400010806.hg.1	6.89	0.0029	IGHV5-51	immunoglobulin heavy variable 5-51	EU433880.1
TC1600007312.hg.1	2.49	<0.0001	IL4R	interleukin 4 receptor	AF421855.1
**Inflammatory Response**					
TC0400011053.hg.1	9.89	0.0001	CXCL10	chemokine (C-X-C motif) ligand 10	NM_001565.3
TC0400011054.hg.1	2.11	0.0029	CXCL11	chemokine (C-X-C motif) ligand 11	NM_020639.2
TC1100009225.hg.1	2.56	0.0068	CXCR5	chemokine (C-X-C motif) receptor 5	NM_032966.2
TC0900008660.hg.1	1.72	0.0027	PTGS1	prostaglandin-endoperoxide synthase 1	NM_001271367.1
TC0500007231.hg.1	2.42	0.0002	PTGER4	prostaglandin E receptor 4 (subtype EP4)	NM_000958.2
TC2200009231.hg.1	1.68	0.0004	MIF	macrophage migration inhibitory factor	NM_002415.1
TC0300011059.hg.1	2.06	0.0002	GPX1	glutathione peroxidase 1	M21304.1
TC1400007752.hg.1	1.94	0.008	GSTZ1	glutathione S-transferase zeta 1	U86529.1
**Cell Adhesion**					
TC1100012686.hg.1	2.7	0.0015	ESAM	endothelial cell adhesion molecule	NM_138961.2
TC1100009611.hg.1	4.79	0.0028	JAM3	junctional adhesion molecule 3	AF448478.1
TC1100006494.hg.1	1.86	0.0023	CD151	CD151 molecule (Raph blood group)	D29963.1
TC0100016357.hg.1	3.28	0.0008	SELP	selectin P	NM_003005.3
TC1700011435.hg.1	1.78	0.0002	ICAM2	intercellular adhesion molecule 2	NM_001099788.1
TC1900009625.hg.1	1.54	0.0052	ICAM3	intercellular adhesion molecule 3	NM_002162.4
TC1300009165.hg.1	1.78	0.0025	PCDH9	protocadherin 9	AF169692.2
TC1700012274.hg.1	7.81	0.0006	ITGB3	integrin beta 3	NM_000212.2
**Blood Coagulation**					
TC1100007938.hg.1	2.09	0.0007	FERMT3	fermitin family member 3	XM_017018398.2
TC0600010709.hg.1	3.2	0.0065	F13A1	coagulation factor XIII, A1 polypeptide	NM_000129.3
TC0100016828.hg.1	1.55	0.0027	F13B	coagulation factor XIII, B polypeptide	NM_001994.2
TC0200015194.hg.1	2.29	0.0008	TFPI	tissue factor pathway inhibitor	NM_006287.5
TC0100008056.hg.1	1.86	0.0063	MPL	MPL proto-oncogene, thrombopoietin receptor	NM_005373.2
TC2200006614.hg.1	2.88	0.0034	GP1BB	glycoprotein Ib (platelet), beta polypeptide	NM_000407.4
TC1000008056.hg.1	2.42	0.0031	VCL	vinculin	M33308.1
TC0700008582.hg.1	1.51	0.0008	SERPINE1	endothelial plasminogen activator inhibitor (PAI-1)	NM_000602.4
**Angiogenesis**					
TC0500012519.hg.1	4.74	0.0005	SPARC	secreted protein, acidic, cysteine-rich (osteonectin)	NM_000602.4
TC0400011053.hg.1	9.89	0.0001	CXCL10	chemokine (C-X-C motif) ligand 10	NM_001565.3
TC1700012468.hg.1	1.74	0.0086	HN1	hematological and neurological expressed 1	AF086910.2
TC1500010429.hg.1	1.91	0.0002	CIB1	calcium and integrin binding 1 (calmyrin)	NM_001277764.1
TC1700012274.hg.1	7.81	0.0006	ITGB3	integrin beta 3	NM_000212.2
TC0700009977.hg.1	1.76	0.0014	PDGFA	platelet-derived growth factor alpha polypeptide	NM_002607.5
TC1900011707.hg.1	1.62	0.0022	GPI	glucose-6-phosphate isomerase	NM_001184722.1
TC0700011770.hg.1	−1.72	0.0003	KRIT1	KRIT1, ankyrin repeat containing	U90268.1
**Positive Regulation of Fibroblast Proliferation**					
TC1200010977.hg.1	1.53	0.0071	CDK4	cyclin-dependent kinase 4	NM_000075.3
TC2200009231.hg.1	1.68	0.0004	MIF	macrophage migration inhibitory factor	NM_002415.1
TC0800008845.hg.1	2.14	0.0029	MYC	v-myc avian myelocytomatosis viral oncogene homolog	NM_002467.5
TC0700009977.hg.1	1.76	0.0014	PDGFA	platelet-derived growth factor alpha polypeptide	NM_002607.5
TC0100015864.hg.1	1.68	0.0021	S100A6	S100 calcium binding protein A6	NM_014624.3
TC0600007847.hg.1	2.68	0.0032	CDKN1A	cyclin-dependent kinase inhibitor 1A (p21, Cip1)	NM_000389
TC0600006873.hg.1	3.9	0.0005	BMP6	bone morphogenetic protein 6	NM_001718.5
**ECM Component and Organization**					
TC0400012213.hg.1	1.53	0.0072	CTSK	cathepsin K	NM_001911.2
TC0500012519.hg.1	4.74	0.0005	SPARC	secreted protein, acidic, cysteine-rich (osteonectin)	J03040.1
TC1900006470.hg.1	1.83	0.0022	BSG	basigin (Ok blood group)	GU557065.1
TC0300007383.hg.1	1.6	0.0035	DAG1	dystroglycan 1 (dystrophin-associated glycoprotein 1)	L19711.1
TC0600008462.hg.1	3.19	0.0037	COL19A1	collagen, type XIX, alpha 1	NM_001858.5
TC0100010863.hg.1	1.67	0.0087	LAMC1	laminin, gamma 1 (formerly LAMB2)	NM_002293.3
TC2000008678.hg.1	2.17	<0.0001	CST3	cystatin C	AH002668.2
**Interferon Alpha/Beta Signaling**					
TC1200012708.hg.1	2.66	0.0007	OAS1	2-5-oligoadenylate synthetase 1	AY730627.1
TC0100015921.hg.1	1.92	<0.0001	ADAR	adenosine deaminase, RNA-specific	NM_015841.4
TC1400010584.hg.1	1.57	0.0015	IRF9	interferon regulatory factor 9	NM_006084.4
TC1500008232.hg.1	2.04	0.0002	ISG20	interferon stimulated exonuclease gene 20kDa	NM_002201.5
TC0100013445.hg.1	3.3	0.0018	IFI6	interferon, alpha-inducible protein 6	NM_002038.3
TC1700007931.hg.1	1.52	0.0078	IFI35	interferon-induced protein 35	NM_001330230.1
TC1000008396.hg.1	6.08	0.0059	IFIT2	interferon-induced protein with tetratricopeptide repeats 2	NM_001547.4
TC1000008401.hg.1	1.65	0.0037	IFIT5	interferon-induced protein with tetratricopeptide repeats 5	NM_012420.2
**IL6-Mediated Signaling Events**					
TC1300008688.hg.1	1.65	0.0072	FOXO1	forkhead box O1	NM_002015.3
TC0700006890.hg.1	12.69	0.0044	IL6	interleukin 6	NM_000600.4
TC1700011903.hg.1	2.03	0.0027	SOCS3	suppressor of cytokine signaling 3	NM_003955.4
TC0800008845.hg.1	2.14	0.0029	MYC	v-myc avian myelocytomatosis viral oncogene homolog	NM_002467.5
**TGF-Beta Receptor Signaling**					
TC0100017258.hg.1	1.75	0.0003	BATF3	basic leucine zipper transcription factor, ATF-like 3	NM_018664.2
TC1800008891.hg.1	2.1	0.0013	BCL2	B-cell CLL/lymphoma 2	NM_000633.2
TC0500013301.hg.1	2.69	0.0015	DAB2	Dab, mitogen-responsive phosphoprotein, homolog 2	NM_001244871.1
TC1300008688.hg.1	1.65	0.0072	FOXO1	forkhead box O1	NM_002015.3
TC1400009426.hg.1	1.85	0.0001	MAX	MYC associated factor X	NM_002382.4
TC0500011418.hg.1	1.55	0.0053	MEF2C	myocyte enhancer factor 2C	NM_002397.4
TC0600013126.hg.1	2.56	0.0005	PTPRK	protein tyrosine phosphatase, receptor type, K	NM_001291983.1
TC0100011533.hg.1	1.93	0.0076	ATF3	activating transcription factor 3	AY426987.1
**PDGFR-Beta Signaling Pathway**					
TC1000007990.hg.1	2.32	0.0017	DDIT4	DNA damage inducible transcript 4	NM_019058.3
TC0300009916.hg.1	1.68	0.0009	HES1	hes family bHLH transcription factor 1	Y07572.1
TC1900010009.hg.1	1.81	0.0021	JUND	jun D proto-oncogene	X56681.1
TC0200008742.hg.1	2.26	0.0001	NCK2	NCK adaptor protein 2	NM_003581.4
TC1500010757.hg.1	1.89	0.0082	RAB11A	RAB11A, member RAS oncogene family	AF000231.1
TC0300008735.hg.1	1.65	0.0014	RAB7A	RAB7A, member RAS oncogene family	NM_004637.5
TC0900010543.hg.1	1.8	0.0034	TLE1	transducin-like enhancer of split 1 (E(sp1) homolog	M99435.1
TC1000008056.hg.1	2.42	0.0031	VCL	vinculin	M33308.1
**VEGF and VEGFR Signaling Network**					
TC0300011899.hg.1	−1.55	0.0038	CBLB	Cbl proto-oncogene B, E3 ubiquitin protein ligase	NM_001321786.1
TC0600011809.hg.1	1.9	<0.0001	CCND3	cyclin D3	NM_001136017.3
TC1200010977.hg.1	1.53	0.0071	CDK4	cyclin-dependent kinase 4	NM_000075.3
TC0500012938.hg.1	2.09	<0.0001	CLTB	clathrin, light chain B	M20470.1
TC0300011059.hg.1	2.06	0.0002	GPX1	glutathione peroxidase 1	M21304.1
TC0200014790.hg.1	1.95	0.0009	GRB14	growth factor receptor bound protein 14	L76687.1
TC0900012167.hg.1	1.81	0.0005	GSN	gelsolin	NM_000177.4
TC0100014014.hg.1	1.53	0.0022	PRDX1	peroxiredoxin 1	NM_002574.3
**Proteoglycan Syndecan-Mediated Signaling Events**					
TC0600006873.hg.1	3.9	0.0005	BMP6	bone morphogenetic protein 6	NM_001718.5
TC1900006470.hg.1	1.83	0.0022	BSG	basigin (Ok blood group)	D45131.1
TC0300007055.hg.1	4.54	0.0005	CTDSPL	CTD small phosphatase like	NM_001008392.1
TC0200014790.hg.1	1.95	0.0009	GRB14	growth factor receptor bound protein 14	L76687.1
TC0200008742.hg.1	2.26	0.0001	NCK2	NCK adaptor protein 2	AF047487.1
TC1100013140.hg.1	1.86	<0.0001	TAF10	TATA-box binding protein associated factor 10	NM_006284.3
TC0500007912.hg.1	−1.58	0.005	ZFYVE16	zinc finger, FYVE domain containing 16	BC030808.1
**Endothelins Signaling Pathway**					
TC1000007990.hg.1	2.32	0.0017	DDIT4	DNA damage inducible transcript 4	NM_019058.3
TC1900009991.hg.1	1.78	0.0055	JAK3	Janus kinase 3	NM_000215.3
TC0300007432.hg.1	1.84	0.0029	MAPKAPK3	mitogen-activated protein kinase-activated protein kinase 3	NM_001243926.1
TC2200008477.hg.1	1.79	0.0044	PATZ1	POZ (BTB) and AT hook containing zinc finger 1	NM_174907.3
TC0600013126.hg.1	2.56	0.0005	PTPRK	protein tyrosine phosphatase, receptor type, K	NM_001135648.2
TC0100016357.hg.1	3.28	0.0008	SELP	selectin P (granule membrane protein 140kDa, antigen CD62)	NM_003005.3
TC1000008056.hg.1	2.42	0.0031	VCL	vinculin	M33308.1
**EGF Receptor (ErbB1) Signaling Pathway**					
TC0300011899.hg.1	−1.55	0.0038	CBLB	Cbl proto-oncogene B, E3 ubiquitin protein ligase	NM_001321786.1
TC1200010977.hg.1	1.53	0.0071	CDK4	cyclin-dependent kinase 4	NM_000075.3
TC0600011635.hg.1	1.87	0.0098	FKBP5	FK506 binding protein 5	NM_004117.3
TC0300007410.hg.1	1.56	0.0005	GNAI2	G protein, alpha inhibiting activity polypeptide 2	NM_002070.3
TC0100010863.hg.1	1.67	0.0087	LAMC1	laminin, gamma 1 (formerly LAMB2)	NM_002293.3
TC0200007446.hg.1	1.67	0.0003	PRKCE	protein kinase C, epsilon	NM_005400.2
TC0700008582.hg.1	1.51	0.0008	SERPINE1	serpin peptidase inhibitor, clade E, member 1	NM_000602.4
TC1800006937.hg.1	1.99	0.0013	TAF4B	TATA box binding protein (TBP)-associated factor, 105kDa	NM_001293725.1

**Table 3:** Selected miRNAs targeted by ncRNA00201.

Total Genes Targeted in Systemic Sclerosis	Disease	References	Genes Targeted in Modules (M)
**hsa-miR-30e-5p**			
22	breast, lung and other cancers	[26]	M1 (UBE2F,SOCS3, UBE2J1) M2 (GNAI2)
**hsa-miR-206**			
20	lung cancer	[27]	M2 (CXCL11) M5 (GCH1)
**hsa-miR-30b-5p**			
20	systemic sclerosis; breast, lung and other cancers	[26,28]	M1 (UBE2F,SOCS3, UBE2J1) M2 (GNAI2)
**hsa-miR-30c-5p**			
20	breast, lung and other cancers	[26]	M1 (UBE2F,SOCS3, UBE2J1) M2 (GNAI2)
**hsa-miR-9-5p**			
20	squamous cell carcinoma	[29]	M2 (CXCL11) M5 (GCH1)
**hsa-miR-30a-5p**			
19	breast and other cancers	[26]	M1 (UBE2F,SOCS3, UBE2J1) M2 (GNAI2)
**hsa-miR-30d-5p**			
19	breast and other cancers	[26]	M1 (UBE2F,SOCS3, UBE2J1) M2 (GNAI2)
**hsa-miR-613**			
19	breast cancer	[30]	M2 (CXCL11) M5 (GCH1)
**hsa-miR-302c-3p**			
17	breast cancer	[31]	M1 (SOCS3, IRF9) M5 (GUCY1A3)
**hsa-miR-302e**			
13	lung cancer	[32]	M1 (IRF9) M5 (GUCY1A3)
**hsa-miR-372-3p**			
12	squamous cell carcinoma	[33]	M1 (IRF9) M5 (GUCY1A3)
**hsa-miR-302a-3p**			
11	breast cancer	[31]	M1 (IRF9) M5 (GUCY1A3)
**hsa-miR-302b-3p**			
11	breast cancer	[31]	M1 (IRF9) M5 (GUCY1A3)
**hsa-miR-302d-3p**			
11	breast cancer	[31]	M1 (IRF9) M5 (GUCY1A3)
**hsa-miR-31-5p**			
11	systemic sclerosis and breast cancer	[34,35]	M3 (KDELR2) M5 (PAX5, SPARC, GCH1)
**hsa-miR-520a-3p**			
11	breast cancer	[36]	M1 (IRF9) M5 (GUCY1A3)
**hsa-miR-520b**			
11	breast, ovarian and other cancers	[37,38]	M1 (IRF9) M5 (GUCY1A3)
**hsa-miR-520c-3p**			
11	breast cancer	[39]	M1 (IRF9) M5 (GUCY1A3)
**hsa-miR-520d-3p**			
11	breast cancer	[40]	M1 (IRF9) M5 (GUCY1A3)
**hsa-miR-520e**			
11	breast cancer	[41]	M1 (IRF9) M5 (GUCY1A3)
**hsa-miR-181d-5p**			
10	leukemia, colon cancer, glioblastoma	[42,43,44]	M1 (CBLB) M5 (PAX5, BCL2) M4 (AP1S3)
**hsa-miR-181a-5p**			
8	leukemia, breast cancer, neuroblastoma	[42,45,46]	M1 (CBLB) M5 (BCL2) M4 (AP1S3)
**hsa-miR-181b-5p**			
8	leukemia, lung cancer, neuroblastoma	[42,46,47]	M1 (CBLB) M5 (BCL2) M4 (AP1S3)
**hsa-miR-181c-5p**			
8	leukemia, sarcoma	[42,48]	M1 (CBLB) M5 (BCL2) M4 (AP1S3)
**hsa-miR-211-5p**			
7	renal cell carcinoma, melanoma, carcinoma, breast cancer	[49,50,51]	M5 (SPARC, BCL2) M6 (PSME1)
**hsa-miR-204-5p**			
7	lung and breast cancer, leukemia, carcinoma	[52,53]	M5 (SPARC, BCL2) M6 (PSME1)

## Supplementary Materials

The following are available online at https://www.mdpi.com/2077-0383/8/3/320/s1, Table S1. Demographic and Clinical features of SSc patients and healthy controls that were enrolled as validation cohort. Table S2. Genes modulated in SSc patients versus healthy subjects. Table S3. Signaling pathways significantly enriched (*p* < 0.05) in genes modulated in SSc patients. Table S4. MicroRNAs and genes that are annotated as targets of ncRNA00201 in StarBase. Table S5. Modulated genes in SSc that are regulated by miRNA targets of ncRNA00201. Table S6. Pathways in which are involved the 138 modulated genes that are targeted by miRNAs modulated by ncRNA00201. Pathways significantly enriched in the 837 genes that were modulated in SSc patients are highlighted in orange. Table S7. Modulated genes in SSc that are included in modules. Genes targeted in the six modules are written in bold characters. Table S8. Signaling pathways in which are involved the five selected genes modulated in SSc. Figure S1. Expression of ncRNA00201 and of selected miRNAs targeted by ncRNA00201 in the 20 SSc patients and healthy controls analyzed in the study and in an expanded panel of SSc patients (20 patients) and healthy controls (20 subjects). Bars indicate SD. Figure S2. Expression of selected genes modulated in SSc patients and regulated by miRNAs targeted by ncRNA00201. Bars indicate SD. Figure S3. Serum levels of selected molecules in SSc patients and in normal subjects. The histograms represent the mean of the results obtained in 20 healthy donors and in 20 SSc patients. Mann-Whitney Test was used to compare the two groups means.
