# Supplementary material for: In Systemic Sclerosis, a Unique Long Non Coding RNA Regulates Genes and Pathways Involved in the Three Main Features of the Disease (Vasculopathy, Fibrosis and Autoimmunity) and in Carcinogenesis
Source: J Clin Med. 2019 Mar 7;8(3):320. doi: 10.3390/jcm8030320 (PMC6462909; doi:10.3390/jcm8030320)
Supplement: Supplementary file 1 [file jcm-08-00320-s001.zip › Supplementary table S1.docx]

| **Demographic and Clinical features of SSc patients and healthy controls of the validation cohort** | | | |
| --- | --- | --- | --- |
| Healthy controls |  | 20 | |
|  | Male/Female | 4/16 | |
|  | Mean age (years) | 54±9 | |
| Patients |  | lSSc | dSSc |
|  |  | 10 | 10 |
|  | Male/Female | 2/8 | 1/9 |
|  | Mean age (years) | 56±10 | 54±9 |
| Laboratory findings | ANA | 8(80%) | 9 (90%) |
|  | Anti-centromere | 5 (50%) | 1 (10%) |
|  | Scl-70 | 0 | 7 (70%) |
| Lung involvement | Interstitial disease | 2 (20%) | 7 (70%) |
|  | Pulmonary arterial hypertension | 1 (10%) | 1 (10%) |
| Skin involvement | mRSS | 9±2 | 16±6 |
|  | Digital ulcers | 2 (20%) | 4 (40%) |
| Video Capillaroscopy | Early | 3 (30%) | 2 (20%) |
|  | Active | 5 (50%) | 5 (50%) |
|  | Late | 2 (20%) | 3 (30%) |
| Kidney involvement |  | 1 (10%) | 1 (10%) |
| Gastro-intestinal involvement |  | 8 (80%) | 10 (100%) |
