# Supplementary figures and images for: In Systemic Sclerosis, a Unique Long Non Coding RNA Regulates Genes and Pathways Involved in the Three Main Features of the Disease (Vasculopathy, Fibrosis and Autoimmunity) and in Carcinogenesis
Source: J Clin Med. 2019 Mar 7;8(3):320. doi: 10.3390/jcm8030320 (PMC6462909; doi:10.3390/jcm8030320)

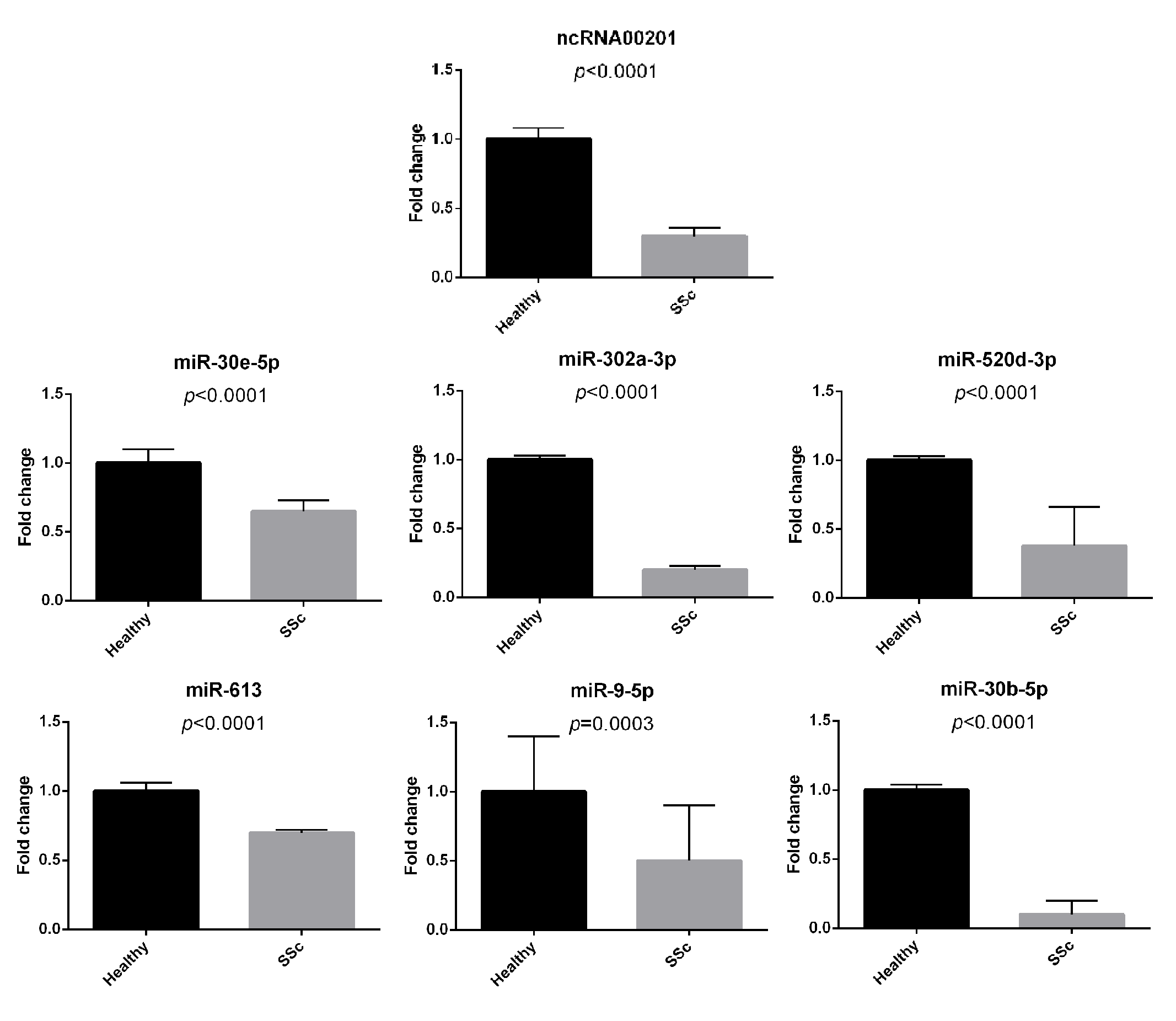

Supplement: Supplementary file 1 [file jcm-08-00320-s001.zip › Supplementary Figure S1 SSC-LONG PCR.tif]

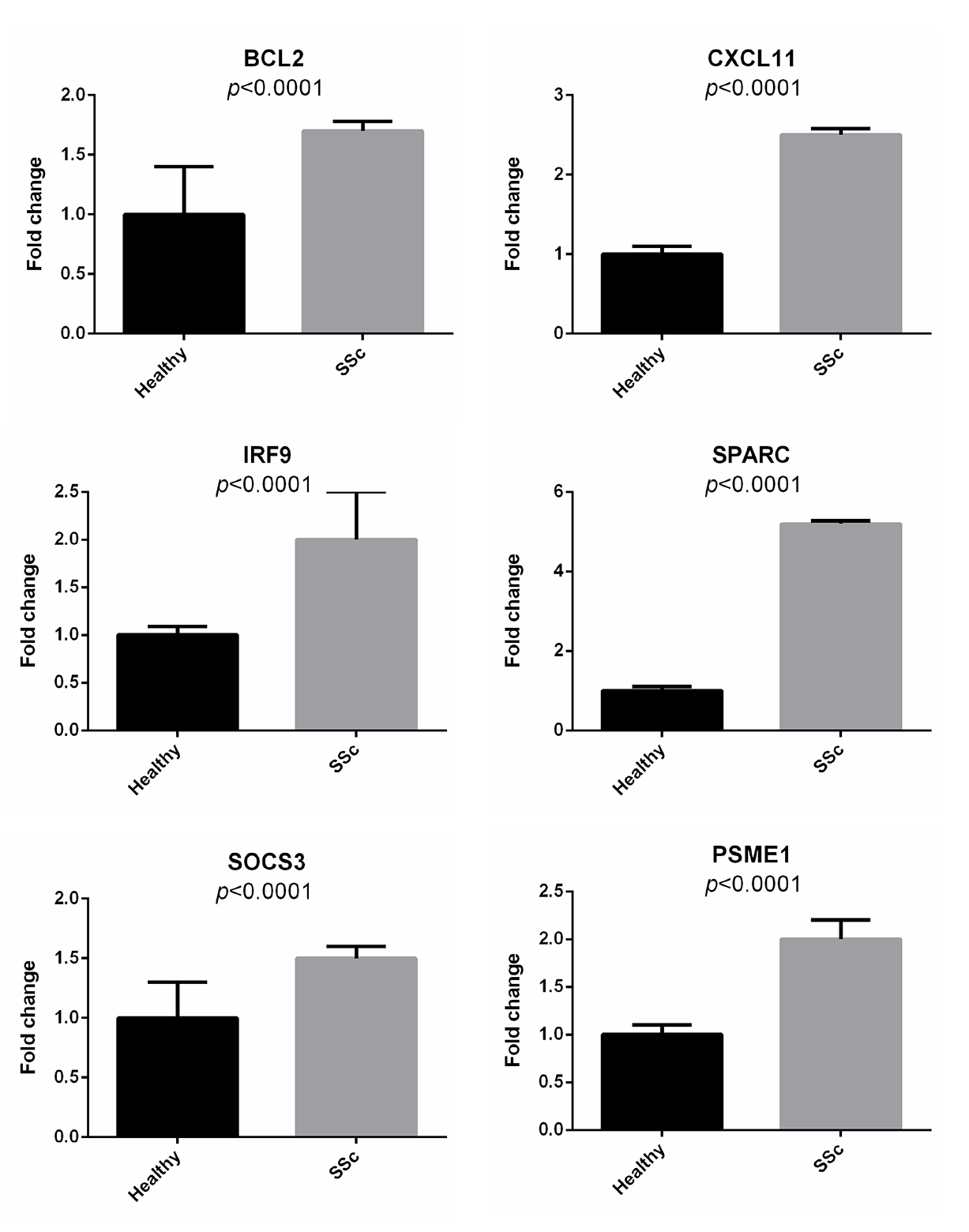

Supplement: Supplementary file 1 [file jcm-08-00320-s001.zip › Supplementary Figure S2 PCR GENI.tif]

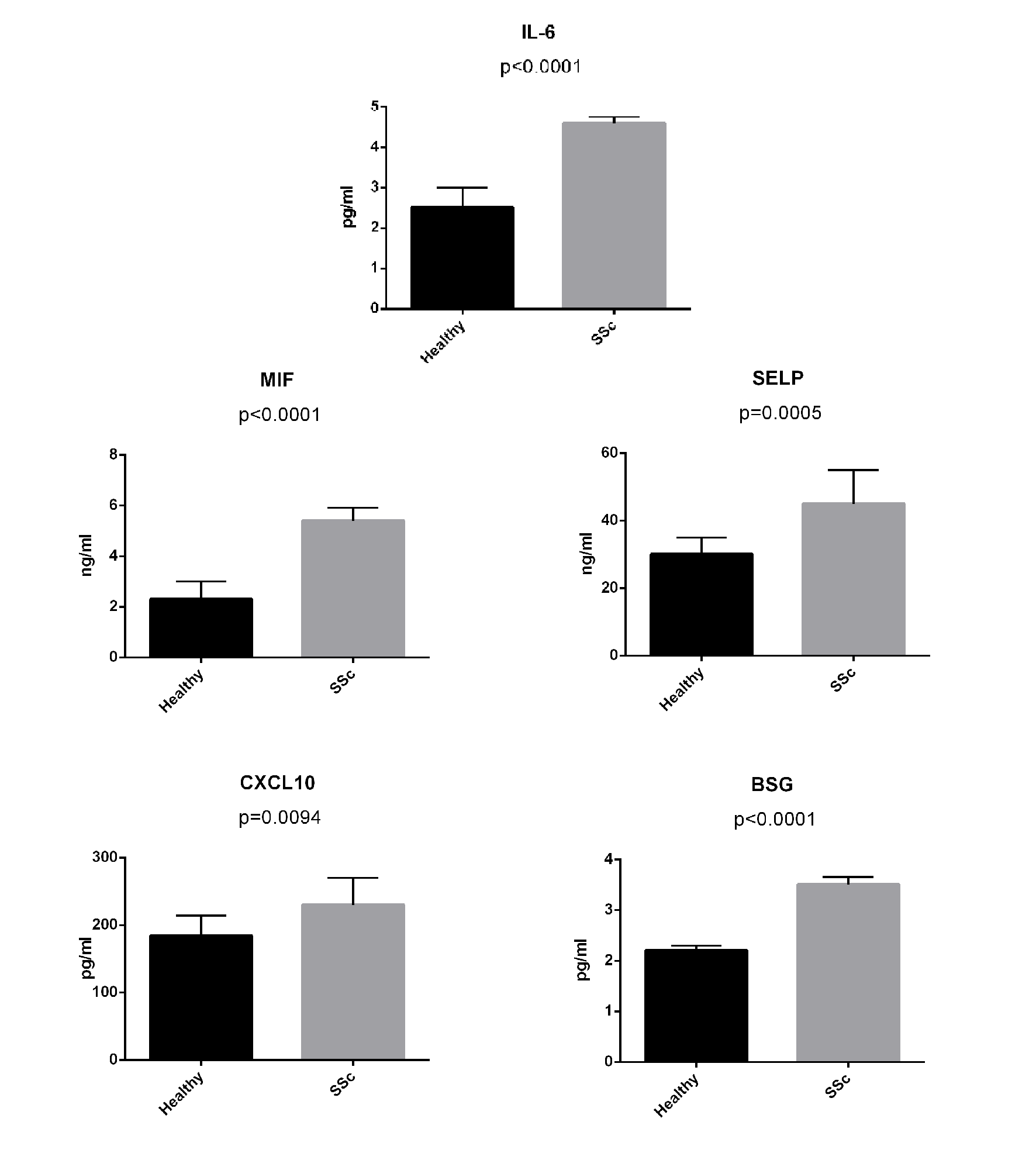

Supplement: Supplementary file 1 [file jcm-08-00320-s001.zip › Supplementary Figure S3 elisa test.tif]
